# 24-Methyl­enecyclo­artanone

**DOI:** 10.1107/S1600536809055123

**Published:** 2010-01-09

**Authors:** Hui-Ping Xiong, Zhi-Jun Wu, Fa-Tang Chen, Wan-Sheng Chen

**Affiliations:** a Department of Mathematics and Physics, Shanghai University of Electric Power, Shanghai 200090, People’s Republic of China; b Department of Pharmacy, Changzheng Hospital, Second Military Medical University, Shanghai 200003, People’s Republic of China

## Abstract

The title compound, C_31_H_50_O, a tetra­cyclic triterpene, was isolated from *Ainsliaea henryi*. The mol­ecule contains a three-membered ring, a five-membered ring, which exhibits an envelope conformation, and three six-membered rings, which adopt chair conformations.

## Related literature

The title compound was first isolated from rice bran oil, see: Ohta & Shimizu (1958 ▶). For its relative stereochemistry, see: Alves *et al.* (2000 ▶); Ohta & Shimizu (1958 ▶). For general background the title compound and the plant *Ainsliaea henryi*, see: Anjaneyulu *et al.* (1999 ▶); Boehme *et al.* (1997 ▶); *Chinese Materia Medica* (2007 ▶); Ei-Dib *et al.* (2004 ▶); Fiechi *et al.* (1966 ▶); Gabrera & Seldes (1995 ▶); Jayasinghe *et al.* (2001 ▶); Kojima *et al.* (1985 ▶); Kolhe *et al.* (1982 ▶); Lao *et al.* (1984 ▶); Lawrie *et al.* (1970 ▶); Li & Xue (1986 ▶); Manoharan *et al.* (2005 ▶); Ohtsu *et al.* (1998 ▶); Schulte *et al.* (1979 ▶); Tachi *et al.* (1971 ▶); Tandon & Rastogi (1976 ▶).
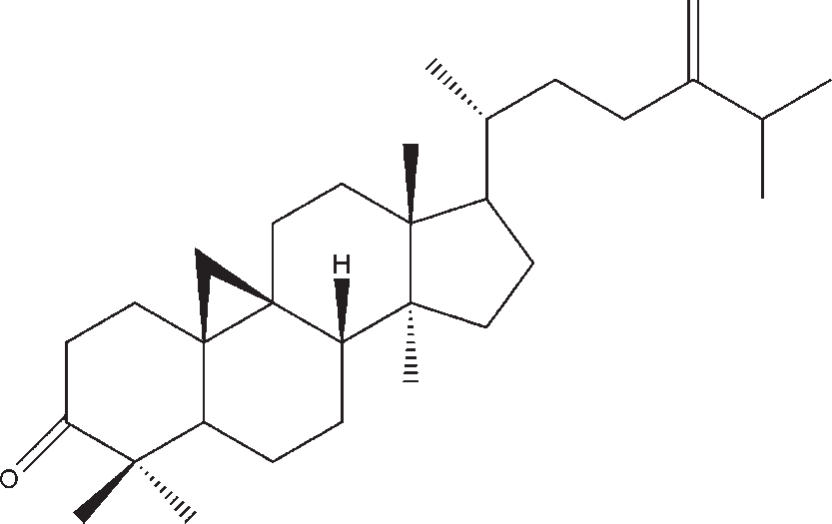


## Experimental

### 

#### Crystal data

C_31_H_50_O
*M* *_r_* = 438.71
Monoclinic, 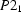

*a* = 9.918 (5) Å
*b* = 10.212 (6) Å
*c* = 14.077 (7) Å
β = 108.542 (6)°
*V* = 1351.7 (12) Å^3^
*Z* = 2
Mo *K*α radiation
μ = 0.06 mm^−1^
*T* = 293 K
0.40 × 0.25 × 0.15 mm

#### Data collection

Bruker SMART APEX CCD area-detector diffractometer
Absorption correction: multi-scan (*SADABS*; Sheldrick, 1996 ▶) *T*_min_ = 0.976, *T*_max_ = 0.991
6145 measured reflections
4360 independent reflections
2901 reflections with *I* > 2σ(*I*)
*R*_int_ = 0.046

#### Refinement

*R*[*F* ^2^ > 2σ(*F* ^2^)] = 0.052
*wR*(*F* ^2^) = 0.133
*S* = 0.91
4360 reflections
296 parameters
1 restraint
H-atom parameters constrained
Δρ_max_ = 0.14 e Å^−3^
Δρ_min_ = −0.20 e Å^−3^

### 

Data collection: *SMART* (Bruker, 2005 ▶); cell refinement: *SAINT* (Bruker, 2005 ▶); data reduction: *SAINT*; program(s) used to solve structure: *SHELXS97* (Sheldrick, 2008 ▶); program(s) used to refine structure: *SHELXL97* (Sheldrick, 2008 ▶); molecular graphics: *SHELXTL* (Sheldrick, 2008 ▶); software used to prepare material for publication: *SHELXTL*.

## Supplementary Material

Crystal structure: contains datablocks I, global. DOI: 10.1107/S1600536809055123/pk2219sup1.cif

Structure factors: contains datablocks I. DOI: 10.1107/S1600536809055123/pk2219Isup2.hkl

Additional supplementary materials:  crystallographic information; 3D view; checkCIF report

## Figures and Tables

**Fig. 1.**
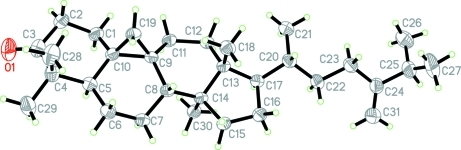
The molecular structure showing the atom-labelling scheme with displacement ellipsoids drawn at the 30% probability level. H atoms are shown as small spheres of arbitrary radius.

**Fig. 2.**
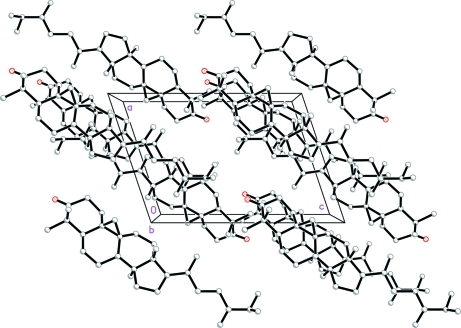
The molecular packing viewed along the b axis.

## supplementary crystallographic information

### Comment

*Ainsliaea henryi* Diels is mainly distributed in south-west of China. The whole plant of *Ainsliaea henryi* has been used in Chinese folk medicine to treat cough, asthma and lumbago (Editorial committee of Chinese Materia Medica, 2007). The chemical constituents of this plant have not all been reported previously. Our chemical investigation of this plant for bioactive components resulted in the isolation of the title compound (I), which was previously first reported isolating from Rice Bran Oil (Ohta & Shimizu, 1958).

The molecular structure is shown in Fig.1. All the bond lengths and angles are within normal ranges. The molecule contains three six-membered rings (A ring atoms C1—C5/C10; B ring atoms C5—C10; C ring atoms C8/C9/C11—C14), a five-member ring (D ring atoms C13—C17), and a three-member ring (E ring C9—C10/C19). Ring A, B and C adopt chair conformations, while ring D exhibits an envelope conformation.

### Experimental

The dry powders (5 kg) of the whole plant of *Ainsliaea henryi* were refluxed for 1 h with 95% ethanol (50*L*) three times. After removal of the ethanol under reduced pressure, the extract was suspended in water and then partitioned with petroleum ether, chloroform, ethyl acetate and n-butanol. The petroleum ether soluble fraction (100 g) was subjected to silica gel column chromatography using gradient elution (petroleum ether/acetone, 30:1 to 10:1, *v*/*v*). 24-methylenecycloartanone was obtained from the fraction eluted by petroleum ether/acetone (20:1). Single crystals suitable for X-ray diffraction analysis were obtained by slow evaporation from acetone after two weeks at room temperature.

### Refinement

The H atoms were located in difference maps and freely refined. As a consequence the absolute configuration of the compound is unknown. The relative stereochemistry of the title compound is known from literature (Alves *et al.*(2000); Ohta & Shimizu (1958)). Its structure was elucidated by chemical methods and by ^1^H, ^13^C, 2D NMR spectroscopy.

### Crystal data

**Table:**

C_31_H_50_O	*F*(000) = 488
*M**_r_* = 438.71	*D*_x_ = 1.078 Mg m^−^^3^
Monoclinic, *P*2_1_	Mo *K*α radiation, λ = 0.71073 Å
*a* = 9.918 (5) Å	Cell parameters from 829 reflections
*b* = 10.212 (6) Å	θ = 2.2–21.1°
*c* = 14.077 (7) Å	µ = 0.06 mm^−^^1^
β = 108.542 (6)°	*T* = 293 K
*V* = 1351.7 (12) Å^3^	Prism, colorless
*Z* = 2	0.40 × 0.25 × 0.15 mm

### Data collection

**Table:**

Bruker SMART APEX CCD area-detector diffractometer	4360 independent reflections
Radiation source: fine-focus sealed tube	2901 reflections with *I* > 2σ(*I*)
graphite	*R*_int_ = 0.046
φ and ω scans	θ_max_ = 26.0°, θ_min_ = 1.5°
Absorption correction: multi-scan (*SADABS*; Sheldrick, 1996)	*h* = −12→12
*T*_min_ = 0.976, *T*_max_ = 0.991	*k* = −10→12
6145 measured reflections	*l* = −17→15

### Refinement

**Table:**

Refinement on *F*^2^	Secondary atom site location: difference Fourier map
Least-squares matrix: full	Hydrogen site location: inferred from neighbouring sites
*R*[*F*^2^ > 2σ(*F*^2^)] = 0.052	H-atom parameters constrained
*wR*(*F*^2^) = 0.133	*w* = 1/[σ^2^(*F*_o_^2^) + (0.0807*P*)^2^] where *P* = (*F*_o_^2^ + 2*F*_c_^2^)/3
*S* = 0.91	(Δ/σ)_max_ = 0.004
4360 reflections	Δρ_max_ = 0.14 e Å^−^^3^
296 parameters	Δρ_min_ = −0.20 e Å^−^^3^
1 restraint	Absolute structure: Flack (1983), 1530 Friedel pairs
0 constraints	Flack parameter: 6 (3)
Primary atom site location: structure-invariant direct methods	

### Special details

**Table:**

Geometry. All e.s.d.'s (except the e.s.d. in the dihedral angle between two l.s. planes) are estimated using the full covariance matrix. The cell e.s.d.'s are taken into account individually in the estimation of e.s.d.'s in distances, angles and torsion angles; correlations between e.s.d.'s in cell parameters are only used when they are defined by crystal symmetry. An approximate (isotropic) treatment of cell e.s.d.'s is used for estimating e.s.d.'s involving l.s. planes.
Refinement. Refinement of *F*^2^ against ALL reflections. The weighted *R*-factor *wR* and goodness of fit *S* are based on *F*^2^, conventional *R*-factors *R* are based on *F*, with *F* set to zero for negative *F*^2^. The threshold expression of *F*^2^ > 2σ(*F*^2^) is used only for calculating *R*-factors(gt) *etc*. and is not relevant to the choice of reflections for refinement. *R*-factors based on *F*^2^ are statistically about twice as large as those based on *F*, and *R*- factors based on ALL data will be even larger.

### Fractional atomic coordinates and isotropic or equivalent isotropic displacement parameters (Å^2^)

**Table:**

	*x*	*y*	*z*	*U*_iso_*/*U*_eq_	
O1	1.1781 (3)	−0.0520 (3)	0.53829 (18)	0.0989 (8)	
C1	1.0700 (3)	−0.0933 (3)	0.7520 (2)	0.0627 (8)	
H1A	1.0112	−0.1649	0.7163	0.075*	
H1B	1.1113	−0.1189	0.8216	0.075*	
C2	1.1884 (3)	−0.0661 (4)	0.7068 (2)	0.0706 (9)	
H2A	1.2560	−0.0056	0.7499	0.085*	
H2B	1.2382	−0.1472	0.7046	0.085*	
C3	1.1341 (3)	−0.0092 (3)	0.6026 (3)	0.0688 (8)	
C4	1.0282 (3)	0.1017 (3)	0.5845 (2)	0.0618 (7)	
C5	0.9150 (3)	0.0692 (3)	0.63678 (18)	0.0535 (6)	
H5	0.8660	−0.0090	0.6022	0.064*	
C6	0.7980 (3)	0.1717 (3)	0.6220 (2)	0.0644 (8)	
H6A	0.8390	0.2540	0.6520	0.077*	
H6B	0.7510	0.1861	0.5511	0.077*	
C7	0.6908 (3)	0.1234 (3)	0.67106 (19)	0.0630 (8)	
H7A	0.6604	0.0355	0.6478	0.076*	
H7B	0.6078	0.1798	0.6517	0.076*	
C8	0.7557 (2)	0.1230 (3)	0.78469 (17)	0.0488 (6)	
H8	0.7796	0.2144	0.8044	0.059*	
C9	0.8977 (2)	0.0462 (3)	0.82016 (17)	0.0487 (6)	
C10	0.9795 (2)	0.0275 (3)	0.74516 (19)	0.0511 (7)	
C11	0.9098 (3)	−0.0640 (3)	0.89571 (19)	0.0567 (7)	
H11A	1.0098	−0.0770	0.9320	0.068*	
H11B	0.8752	−0.1438	0.8586	0.068*	
C12	0.8305 (3)	−0.0463 (3)	0.97370 (18)	0.0551 (7)	
H12A	0.7763	−0.1252	0.9746	0.066*	
H12B	0.9003	−0.0367	1.0396	0.066*	
C13	0.7297 (2)	0.0710 (3)	0.95408 (16)	0.0457 (6)	
C14	0.6517 (2)	0.0792 (3)	0.83892 (17)	0.0454 (6)	
C15	0.5341 (3)	0.1789 (3)	0.83282 (19)	0.0555 (7)	
H15A	0.5688	0.2675	0.8315	0.067*	
H15B	0.4534	0.1648	0.7731	0.067*	
C16	0.4922 (3)	0.1564 (3)	0.92712 (19)	0.0596 (7)	
H16A	0.3980	0.1181	0.9096	0.072*	
H16B	0.4915	0.2389	0.9612	0.072*	
C17	0.6035 (2)	0.0623 (3)	0.99590 (17)	0.0492 (6)	
H17	0.5645	−0.0264	0.9813	0.059*	
C18	0.8169 (3)	0.1938 (3)	0.9943 (2)	0.0617 (7)	
H18A	0.8885	0.2042	0.9624	0.092*	
H18B	0.8615	0.1855	1.0654	0.092*	
H18C	0.7555	0.2689	0.9805	0.092*	
C19	1.0290 (3)	0.1257 (3)	0.82928 (19)	0.0608 (7)	
H19A	1.1144	0.1051	0.8841	0.073*	
H19B	1.0165	0.2179	0.8120	0.073*	
C20	0.6255 (3)	0.0873 (3)	1.10733 (18)	0.0570 (7)	
H20	0.6570	0.1781	1.1224	0.068*	
C21	0.7383 (3)	−0.0022 (4)	1.1761 (2)	0.0780 (10)	
H21A	0.7108	−0.0920	1.1612	0.117*	
H21B	0.7469	0.0161	1.2446	0.117*	
H21C	0.8280	0.0129	1.1655	0.117*	
C22	0.4822 (3)	0.0714 (3)	1.12670 (18)	0.0603 (7)	
H22A	0.4172	0.1368	1.0874	0.072*	
H22B	0.4436	−0.0138	1.1018	0.072*	
C23	0.4841 (3)	0.0832 (4)	1.23481 (19)	0.0675 (8)	
H23A	0.5483	0.0173	1.2742	0.081*	
H23B	0.5230	0.1681	1.2600	0.081*	
C24	0.3429 (3)	0.0683 (3)	1.2521 (2)	0.0671 (8)	
C25	0.3436 (4)	0.0745 (4)	1.3605 (2)	0.0851 (10)	
H25	0.2496	0.0427	1.3590	0.102*	
C26	0.4480 (4)	−0.0188 (4)	1.4282 (2)	0.0938 (11)	
H26A	0.4323	−0.0225	1.4920	0.141*	
H26B	0.5431	0.0109	1.4373	0.141*	
H26C	0.4354	−0.1045	1.3986	0.141*	
C27	0.3530 (6)	0.2084 (4)	1.3999 (3)	0.1210 (16)	
H27A	0.2708	0.2572	1.3620	0.181*	
H27B	0.4372	0.2498	1.3945	0.181*	
H27C	0.3573	0.2057	1.4689	0.181*	
C28	1.1138 (4)	0.2263 (3)	0.6265 (3)	0.0837 (10)	
H28A	1.0498	0.2988	0.6200	0.126*	
H28B	1.1784	0.2445	0.5899	0.126*	
H28C	1.1666	0.2133	0.6960	0.126*	
C29	0.9553 (4)	0.1175 (4)	0.4711 (2)	0.0858 (10)	
H29A	0.8986	0.0415	0.4453	0.129*	
H29B	1.0263	0.1273	0.4385	0.129*	
H29C	0.8956	0.1938	0.4587	0.129*	
C30	0.5813 (3)	−0.0514 (3)	0.79658 (19)	0.0564 (7)	
H30A	0.6532	−0.1133	0.7946	0.085*	
H30B	0.5172	−0.0379	0.7300	0.085*	
H30C	0.5295	−0.0845	0.8386	0.085*	
C31	0.2222 (3)	0.0485 (5)	1.1819 (3)	0.0985 (13)	
H31A	0.2195	0.0427	1.1154	0.118*	
H31B	0.1389	0.0403	1.1983	0.118*	

### Atomic displacement parameters (Å^2^)

**Table:**

	*U*^11^	*U*^22^	*U*^33^	*U*^12^	*U*^13^	*U*^23^
O1	0.1252 (19)	0.092 (2)	0.1038 (17)	0.0216 (16)	0.0714 (15)	0.0033 (16)
C1	0.0527 (15)	0.068 (2)	0.0665 (17)	0.0111 (14)	0.0177 (13)	0.0094 (15)
C2	0.0561 (16)	0.083 (2)	0.0737 (19)	0.0086 (16)	0.0222 (14)	−0.0040 (18)
C3	0.0722 (19)	0.062 (2)	0.083 (2)	−0.0045 (16)	0.0394 (17)	−0.0080 (17)
C4	0.0677 (16)	0.059 (2)	0.0637 (17)	−0.0049 (15)	0.0278 (13)	−0.0020 (15)
C5	0.0566 (14)	0.0481 (16)	0.0532 (14)	−0.0033 (14)	0.0139 (11)	−0.0010 (13)
C6	0.0673 (17)	0.066 (2)	0.0598 (16)	0.0105 (15)	0.0198 (13)	0.0175 (15)
C7	0.0539 (15)	0.076 (2)	0.0544 (15)	0.0136 (14)	0.0109 (12)	0.0157 (15)
C8	0.0470 (13)	0.0434 (14)	0.0522 (14)	0.0029 (11)	0.0106 (11)	0.0036 (12)
C9	0.0424 (13)	0.0552 (18)	0.0434 (13)	0.0042 (12)	0.0066 (10)	0.0017 (12)
C10	0.0418 (13)	0.0554 (17)	0.0538 (15)	0.0041 (12)	0.0122 (11)	0.0004 (12)
C11	0.0500 (14)	0.0578 (18)	0.0596 (16)	0.0163 (13)	0.0137 (12)	0.0101 (14)
C12	0.0539 (15)	0.0563 (18)	0.0501 (15)	0.0095 (13)	0.0095 (12)	0.0075 (13)
C13	0.0418 (12)	0.0434 (14)	0.0477 (13)	0.0023 (12)	0.0086 (10)	0.0003 (12)
C14	0.0409 (11)	0.0410 (14)	0.0489 (13)	0.0029 (11)	0.0067 (10)	−0.0024 (12)
C15	0.0498 (14)	0.0590 (18)	0.0554 (15)	0.0119 (13)	0.0135 (12)	0.0026 (13)
C16	0.0591 (15)	0.0576 (19)	0.0606 (15)	0.0128 (13)	0.0169 (12)	0.0001 (14)
C17	0.0498 (13)	0.0439 (15)	0.0503 (13)	0.0015 (12)	0.0110 (11)	−0.0017 (13)
C18	0.0627 (17)	0.0607 (19)	0.0582 (16)	−0.0092 (14)	0.0145 (13)	−0.0077 (14)
C19	0.0494 (14)	0.072 (2)	0.0584 (16)	−0.0089 (14)	0.0129 (12)	−0.0050 (15)
C20	0.0608 (15)	0.0550 (18)	0.0520 (14)	−0.0003 (14)	0.0135 (12)	−0.0047 (13)
C21	0.0720 (18)	0.103 (3)	0.0548 (17)	0.0160 (18)	0.0145 (14)	0.0079 (17)
C22	0.0714 (16)	0.0582 (17)	0.0521 (15)	0.0002 (15)	0.0206 (12)	−0.0068 (14)
C23	0.0748 (17)	0.072 (2)	0.0535 (15)	0.0040 (16)	0.0165 (13)	−0.0042 (16)
C24	0.0770 (19)	0.067 (2)	0.0611 (17)	0.0106 (18)	0.0266 (15)	−0.0006 (16)
C25	0.099 (2)	0.104 (3)	0.0598 (19)	0.011 (2)	0.0346 (17)	0.004 (2)
C26	0.115 (3)	0.095 (3)	0.067 (2)	−0.010 (2)	0.0237 (19)	0.013 (2)
C27	0.209 (5)	0.088 (3)	0.083 (3)	0.015 (3)	0.071 (3)	−0.014 (2)
C28	0.092 (2)	0.063 (2)	0.110 (3)	−0.0162 (19)	0.051 (2)	0.000 (2)
C29	0.107 (2)	0.089 (3)	0.071 (2)	0.007 (2)	0.0418 (18)	0.0105 (19)
C30	0.0497 (14)	0.0582 (18)	0.0587 (16)	−0.0042 (13)	0.0134 (12)	−0.0114 (14)
C31	0.074 (2)	0.151 (4)	0.072 (2)	0.004 (2)	0.0248 (18)	0.000 (2)

### Geometric parameters (Å, °)

**Table:**

O1—C3	1.205 (3)	C16—H16A	0.9700
C1—C10	1.511 (4)	C16—H16B	0.9700
C1—C2	1.528 (4)	C17—C20	1.535 (4)
C1—H1A	0.9700	C17—H17	0.9800
C1—H1B	0.9700	C18—H18A	0.9600
C2—C3	1.509 (4)	C18—H18B	0.9600
C2—H2A	0.9700	C18—H18C	0.9600
C2—H2B	0.9700	C19—H19A	0.9700
C3—C4	1.511 (4)	C19—H19B	0.9700
C4—C29	1.537 (4)	C20—C21	1.528 (4)
C4—C28	1.539 (4)	C20—C22	1.539 (4)
C4—C5	1.562 (4)	C20—H20	0.9800
C5—C10	1.516 (4)	C21—H21A	0.9600
C5—C6	1.527 (4)	C21—H21B	0.9600
C5—H5	0.9800	C21—H21C	0.9600
C6—C7	1.522 (4)	C22—C23	1.521 (4)
C6—H6A	0.9700	C22—H22A	0.9700
C6—H6B	0.9700	C22—H22B	0.9700
C7—C8	1.523 (4)	C23—C24	1.504 (4)
C7—H7A	0.9700	C23—H23A	0.9700
C7—H7B	0.9700	C23—H23B	0.9700
C8—C14	1.532 (3)	C24—C31	1.303 (4)
C8—C9	1.549 (3)	C24—C25	1.524 (4)
C8—H8	0.9800	C25—C27	1.467 (6)
C9—C19	1.505 (4)	C25—C26	1.504 (5)
C9—C11	1.527 (4)	C25—H25	0.9800
C9—C10	1.534 (4)	C26—H26A	0.9600
C10—C19	1.510 (4)	C26—H26B	0.9600
C11—C12	1.551 (4)	C26—H26C	0.9600
C11—H11A	0.9700	C27—H27A	0.9600
C11—H11B	0.9700	C27—H27B	0.9600
C12—C13	1.528 (4)	C27—H27C	0.9600
C12—H12A	0.9700	C28—H28A	0.9600
C12—H12B	0.9700	C28—H28B	0.9600
C13—C18	1.526 (4)	C28—H28C	0.9600
C13—C17	1.546 (3)	C29—H29A	0.9600
C13—C14	1.561 (3)	C29—H29B	0.9600
C14—C15	1.530 (3)	C29—H29C	0.9600
C14—C30	1.534 (4)	C30—H30A	0.9600
C15—C16	1.529 (4)	C30—H30B	0.9600
C15—H15A	0.9700	C30—H30C	0.9600
C15—H15B	0.9700	C31—H31A	0.9300
C16—C17	1.549 (3)	C31—H31B	0.9300
			
C10—C1—C2	110.2 (2)	C17—C16—H16A	110.2
C10—C1—H1A	109.6	C15—C16—H16B	110.2
C2—C1—H1A	109.6	C17—C16—H16B	110.2
C10—C1—H1B	109.6	H16A—C16—H16B	108.5
C2—C1—H1B	109.6	C20—C17—C13	120.69 (19)
H1A—C1—H1B	108.1	C20—C17—C16	112.2 (2)
C3—C2—C1	113.0 (2)	C13—C17—C16	103.2 (2)
C3—C2—H2A	109.0	C20—C17—H17	106.7
C1—C2—H2A	109.0	C13—C17—H17	106.7
C3—C2—H2B	109.0	C16—C17—H17	106.7
C1—C2—H2B	109.0	C13—C18—H18A	109.5
H2A—C2—H2B	107.8	C13—C18—H18B	109.5
O1—C3—C2	119.3 (3)	H18A—C18—H18B	109.5
O1—C3—C4	122.9 (3)	C13—C18—H18C	109.5
C2—C3—C4	117.8 (3)	H18A—C18—H18C	109.5
C3—C4—C29	109.1 (3)	H18B—C18—H18C	109.5
C3—C4—C28	106.6 (2)	C9—C19—C10	61.19 (17)
C29—C4—C28	109.4 (3)	C9—C19—H19A	117.6
C3—C4—C5	109.0 (2)	C10—C19—H19A	117.6
C29—C4—C5	109.9 (2)	C9—C19—H19B	117.6
C28—C4—C5	112.7 (2)	C10—C19—H19B	117.6
C10—C5—C6	112.9 (2)	H19A—C19—H19B	114.8
C10—C5—C4	113.4 (2)	C21—C20—C17	112.7 (2)
C6—C5—C4	114.8 (2)	C21—C20—C22	110.9 (2)
C10—C5—H5	104.8	C17—C20—C22	108.92 (19)
C6—C5—H5	104.8	C21—C20—H20	108.1
C4—C5—H5	104.8	C17—C20—H20	108.1
C7—C6—C5	109.1 (2)	C22—C20—H20	108.1
C7—C6—H6A	109.9	C20—C21—H21A	109.5
C5—C6—H6A	109.9	C20—C21—H21B	109.5
C7—C6—H6B	109.9	H21A—C21—H21B	109.5
C5—C6—H6B	109.9	C20—C21—H21C	109.5
H6A—C6—H6B	108.3	H21A—C21—H21C	109.5
C6—C7—C8	110.7 (2)	H21B—C21—H21C	109.5
C6—C7—H7A	109.5	C23—C22—C20	116.8 (2)
C8—C7—H7A	109.5	C23—C22—H22A	108.1
C6—C7—H7B	109.5	C20—C22—H22A	108.1
C8—C7—H7B	109.5	C23—C22—H22B	108.1
H7A—C7—H7B	108.1	C20—C22—H22B	108.1
C7—C8—C14	113.4 (2)	H22A—C22—H22B	107.3
C7—C8—C9	112.2 (2)	C24—C23—C22	116.0 (2)
C14—C8—C9	112.2 (2)	C24—C23—H23A	108.3
C7—C8—H8	106.1	C22—C23—H23A	108.3
C14—C8—H8	106.1	C24—C23—H23B	108.3
C9—C8—H8	106.1	C22—C23—H23B	108.3
C19—C9—C11	117.6 (2)	H23A—C23—H23B	107.4
C19—C9—C10	59.54 (17)	C31—C24—C23	124.8 (3)
C11—C9—C10	116.5 (2)	C31—C24—C25	118.5 (3)
C19—C9—C8	115.3 (2)	C23—C24—C25	116.7 (3)
C11—C9—C8	117.4 (2)	C27—C25—C26	113.7 (3)
C10—C9—C8	117.8 (2)	C27—C25—C24	113.3 (3)
C19—C10—C1	117.0 (2)	C26—C25—C24	113.0 (3)
C19—C10—C5	122.1 (2)	C27—C25—H25	105.2
C1—C10—C5	110.2 (2)	C26—C25—H25	105.2
C19—C10—C9	59.27 (17)	C24—C25—H25	105.2
C1—C10—C9	119.4 (2)	C25—C26—H26A	109.5
C5—C10—C9	120.8 (2)	C25—C26—H26B	109.5
C9—C11—C12	117.5 (2)	H26A—C26—H26B	109.5
C9—C11—H11A	107.9	C25—C26—H26C	109.5
C12—C11—H11A	107.9	H26A—C26—H26C	109.5
C9—C11—H11B	107.9	H26B—C26—H26C	109.5
C12—C11—H11B	107.9	C25—C27—H27A	109.5
H11A—C11—H11B	107.2	C25—C27—H27B	109.5
C13—C12—C11	114.4 (2)	H27A—C27—H27B	109.5
C13—C12—H12A	108.7	C25—C27—H27C	109.5
C11—C12—H12A	108.7	H27A—C27—H27C	109.5
C13—C12—H12B	108.7	H27B—C27—H27C	109.5
C11—C12—H12B	108.7	C4—C28—H28A	109.5
H12A—C12—H12B	107.6	C4—C28—H28B	109.5
C18—C13—C12	108.44 (19)	H28A—C28—H28B	109.5
C18—C13—C17	109.7 (2)	C4—C28—H28C	109.5
C12—C13—C17	116.6 (2)	H28A—C28—H28C	109.5
C18—C13—C14	112.1 (2)	H28B—C28—H28C	109.5
C12—C13—C14	108.02 (19)	C4—C29—H29A	109.5
C17—C13—C14	101.81 (16)	C4—C29—H29B	109.5
C15—C14—C8	113.5 (2)	H29A—C29—H29B	109.5
C15—C14—C30	108.2 (2)	C4—C29—H29C	109.5
C8—C14—C30	110.8 (2)	H29A—C29—H29C	109.5
C15—C14—C13	102.19 (19)	H29B—C29—H29C	109.5
C8—C14—C13	110.07 (18)	C14—C30—H30A	109.5
C30—C14—C13	111.8 (2)	C14—C30—H30B	109.5
C16—C15—C14	105.3 (2)	H30A—C30—H30B	109.5
C16—C15—H15A	110.7	C14—C30—H30C	109.5
C14—C15—H15A	110.7	H30A—C30—H30C	109.5
C16—C15—H15B	110.7	H30B—C30—H30C	109.5
C14—C15—H15B	110.7	C24—C31—H31A	120.0
H15A—C15—H15B	108.8	C24—C31—H31B	120.0
C15—C16—C17	107.4 (2)	H31A—C31—H31B	120.0
C15—C16—H16A	110.2		
